# Episodic memory in animals?

**DOI:** 10.1515/tnsci-2025-0383

**Published:** 2025-11-21

**Authors:** Thomas R. Zentall

**Affiliations:** Department of Psychology, University of Kentucky, Lexington, KY, 40506-0044, United States

**Keywords:** episodic memory, unexpected question, incidental learning, what–where–when, animals

## Abstract

Episodic memory refers to the recall of memories concerned with unique, personal past experiences. It has been differentiated from semantic or rule learning that may be associatively learned. Episodic memory has the quality of mentally traveling back in time to recover a memory. Episodic memory is believed to be related to language and consciousness, and for this reason, has been thought to be unique to humans. Having episodic memory generally allows one to describe what happened, where it happened, and when it happened. For some time, research has focused on these three properties, and research with animals indicates that several species do have the capacity to behaviorally report the what, where, and when of an event. But such evidence is not sufficient to conclude that animals have episodic memory. Instead, to better distinguish episodic memory from semantic or rule learning, an organism should be able to respond appropriately to an unexpected question about an event that was incidentally rather than explicitly encoded. Using this criterion, there is growing evidence that several species do have such an ability. Furthermore, it is proposed that episodic memory serves to enable future planning, and animals show some evidence for that as well.

Human memory is thought to be divided into two main categories: (1) nondeclarative memory consisting of procedural memory (knowing how something is done), Pavlovian conditioning, and priming; and (2) declarative memory (knowing that) consisting of semantic or factual memory and episodic memory (memory for experienced events). According to Tulving [1], episodic memory refers to the acquisition and recall of memories concerned with unique, personal past experiences. It is accepted that humans have episodic memory because as individuals we are personally conscious of the process of retrieving events from the past, but it is not so easy to study episodic memory in others even other people.

Although several methods for studying human episodic memory have been developed, the best evidence for a distinctive memory system for personal experiences in humans comes from research on brain-injured individuals who have lost the ability to remember personal past experiences [2]. If autonoetic consciousness [3] or self-knowledge is necessary for the demonstration of episodic memory, it may not be possible to demonstrate such a capacity in animals because, in the absence of language, it is not clear what would constitute evidence of consciousness. According to the Bischof–Kohler hypothesis [4], nonhuman animals cannot anticipate a future event and take appropriate action when that event involves satisfaction of a need not currently present. Thus, animals should not be capable of having episodic memory or be able to make future plans.

## 1 What is the necessary evidence for episodic memory in animals?

Intuitively, one might consider that animals must have episodic memory because they act as if they remember past experiences. For example, they are able to retain what they learned the day before. If an animal could talk, however, it might be clear whether or not they have episodic memory. For instance, the day after a rat had been trained to press a lever, and as it is getting ready to press the lever again, one could ask it what it is going to do and why. The rat might answer that it is going to press the lever because it thinks (or knows) a pellet will appear. However, it might not remember ever having pressed the lever before. That is, it might *know* that pressing the lever produces food without having a memory for having done so before.

Tulving [1] originally proposed that episodic memory “receives and stores information about temporally dated episodes or events, and temporal-spatial relations among those events” (p. 385). This definition can be interpreted to mean that episodic memory provides information about what event occurred, as well as where and when it happened. He later described episodic memory in terms of mental time travel, traveling back in time to reexperience an earlier episode (a process he referred to as *autonoetic memory*). Because evidence for episodic memory is thought to require language, it is presumed to be beyond the capacity of nonhuman animals [5]. However, some investigators have used Tulving’s [1] earlier description of episodic memory to ask if a nonverbal animal can remember what occurred, where it occurred, and when it occurred.

## 2 The what, where, and when of episodic memory

Several studies with primates have found that they have good long-term memory for the what and where (or who) of events that they have experienced [6,7]. However, it is assumed that *where* something (*what*) can be found does not require episodic memory because it can be accounted for by associative (rule) learning. For example, one can train a rat to press the left lever (where) for water (what) and to press the right lever (where) for food (what). Those would be considered learned associative responses. Thus, it would appear that the key to episodic memory is the *when* of a memory.

Clayton and Dickinson [8] conducted a now classic experiment with scrub jays in which the jays first learned that their preferred reward, wax worms, degrades over time and becomes unpalatable, but their less preferred peanuts do not. The jays were then allowed to cache wax worms on one side of a distinctive tray and peanuts on the other side. When they were then allowed to recover food 4 h later, relative to the peanuts, they inspected the location where they had cached the worms significantly more often than when they were allowed to recover the food 124 h later. That is, not only did the jays remember where they cached their preferred food (what) but importantly they also remembered when they had cached it (see also [9,10]). There is also evidence that two other food-caching species, magpies [11] and black-capped chickadees [12], pass this what–where–when test.

A different approach to the what–where–when test was demonstrated in rats [13]. An eight-arm radial maze was used in which rats were allowed to enter a random four baited arms; three were baited with rat chow, while one was baited with a preferred chocolate chip. Two minutes later, in a standard memory test, the rats were replaced on the maze, and all arms were open with the originally unbaited arms now baited. An experimental trial was conducted either in the morning or in the afternoon. If it was a morning trial, when the rats were tested, the arm in which they had experienced chocolate was replenished with chocolate. If it was an afternoon trial, however, the chocolate was not replenished. The critical measure in the experiment was how often the rats revisited the chocolate location when tested in the morning and when they were tested in the afternoon. Results indicated that the rats revisited the chocolate location significantly more often in the morning when the chocolate arm was replenished. Thus, the rats remembered the chocolate (what), where the chocolate had been, and when the trial was run (morning or afternoon). Other experiments found comparable results and also ruled out alternative accounts [14,15,16,17].

Another aspect of episodic memory is that the what–where–when of it is presumed to be recovered as an integrated unit [18]. To test for the notion of an integrated unit, pigeons were trained on a task in which they had to learn the identity of a stimulus (what), the location of a stimulus (where), and how long ago the stimulus had been presented (when). The pigeons learned to respond correctly to each of those features of the stimulus but they did not appear to process those three features together as a unit. When, on a given trial, they were asked about two of those features in succession, their response was not consistent with an integrated memory of the stimulus presented. In other experiments, however, evidence for the integration of what, where, and when has been found in rats [19], scrub jays [20], and monkeys [21]. In these experiments, however, the fact that the what, where, and when features were trained independently may raise questions as to whether the results can be interpreted as having developed episodic memory.

One problem with the what, where, and when procedure is, because the animals are trained to remember those aspects of the task, they may learn to update information as it occurs. Therefore, they may *know* what food it is, where it is, and, in the case of the scrub jays, for example, whether it is fresh or not. That is, they may learn it associatively [22].

Furthermore, although evidence for the what, where, and when of memory would seem to be important if a memory is thought to be episodic, it can be argued that this criterion is not sufficient, in principle, as evidence of episodic memory. For example, I may know that the Declaration of Independence (what) was signed in Philadelphia (where) on July 4, 1776 (when), although clearly that is not an episodic memory. Thus, what, where, and when is not sufficient. Conversely, what, where, and when may not even be necessary. For example, I may have a vivid episodic memory of having met someone before, but I may be unable to recall when or even where the meeting took place [22].

Language-trained primates offer a unique approach to the study of episodic memory in animals [23]. In that research, a chimpanzee could see food being placed outside the fence of the enclosure, and then the chimpanzee was moved back inside. Later, when a different caretaker arrived, the chimpanzee pointed to the lexicon (symbol for the food) and pointed in the direction of the food to indicate her memory for the past event. As Schwartz et al. [7] noted, however, the chimpanzee’s memory need not have been past-oriented. It is possible that she updated her memory about spatial landmarks and their contents and did not need to mentally refer back to the event of watching the food being hidden.

Tulving [3] later recognized that the what–where–when definition was not adequate and proposed that episodic memory had the quality of *autonoesis* or self-knowledge. That is the kind of conscious awareness that characterizes conscious recollection. As Boyle notes [22], however, these terms cannot be reduced to or captured in terms of the representational content of memory. That is, autonoesis cannot easily be measured. For human episodic memory, although one can conceive of it as the retrieval of the details of an event that are integrated into a single representation, such a definition would not be of great value in trying to identify episodic memory in a nonverbal animal.

## 3 The unexpected question about an incidental experience

When information is explicitly encoded for use in an expected memory test, explicitly encoded information may generate a learned association. When the opportunity to perform the action occurs, an animal may merely execute the action without having to remember back in time to the earlier event. In such a case, the remembered action can occur without the need to retrieve an episodic memory. Although episodic memories may be explicitly learned, it is not clear that such memories can be distinguished from semantic or rule learning, especially in nonverbal animals. Thus, evidence for episodic memory should be generated by an experience that does not involve an explicitly trained association.

One approach to solving this problem comes from a classic way that episodic memory can be illustrated to students. For example, one can ask a student what they had for dinner last night. If the response was “it was pizza because last night was Monday and I always have pizza Monday night,” one would describe that as a semantic memory because it is simply a known bit of information that they would not have to retrieved from a personal experience. Alternatively, however, more typically the response involves mentally traveling back in time to recover the memory of the event. Interestingly, when one does that, one often recovers not only the meal that one has had, but also the activity leading up to the dinner and the context in which dinner took place. That is, one is often able to recreate the entire episode. In the case of such a response, one would likely describe that as an episodic memory because in addition to the answer to the question, one can often relate the antecedent conditions and the context in which the dinner took place. The question is, is there some way to convert the what-did-you-have-for-dinner question into a procedure that could be used with a non-verbal animal. What is critical about the question is that the answer is not something that would be typically committed to memory. That is, the information should be *incidentally learned*, and its retrieval should occur by way of an *unexpected question*.

## 4 The answer to an unexpected question in pigeons

**Figure 1:**
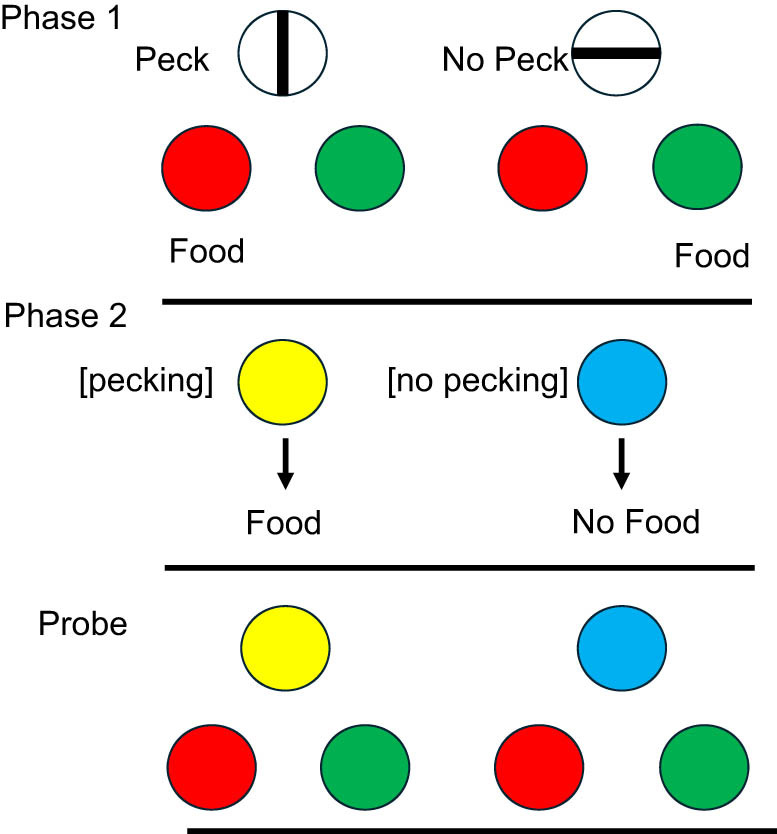
Design of pigeon episodic memory experiment: “Did you peck or not?”: In Phase 1, pigeons were trained to peck at the vertical line and to refrain from pecking at the horizontal line to obtain the red and green comparison stimuli. If they had pecked, red was correct. If they refrained from pecking, green was correct. In Phase 2, Yellow was followed by food [pecking occurred]. Blue was followed by the absence of food [pecking did not occur]. On probe trials, yellow and blue were followed by a choice between red and green [23].

Zentall et al. [24] attempted to assess episodic memory in pigeons using something like the unexpected question concerning an incidental memory. However, before giving the pigeons an experience that they would have to retrieve, the pigeons first had to be trained with a procedure that can be thought of as giving them the opportunity to respond to a question. One can think of this as giving the pigeons a rudimentary language. Thus, to be able to communicate with the pigeon, in Phase 1 of their procedure, the pigeons were trained on a conditional discrimination (Figure 1). When vertical lines were presented as a sample, the first peck to the sample after 4 s terminated the sample and presented red and green lights to the left and right. A peck to the red light was rewarded. When horizontal lines were presented as the sample, the pigeon was required to refrain from pecking for 4 s, after which red and green lights appeared on the left and right, and a peck to the green light was rewarded. Thus, one could interpret the red and green lights as serving as the question, “What did you just do, did you just peck or did you just refrain from pecking?”

In Phase 2 of the experiment, the pigeons were exposed to episodic events. On some trials, the pigeons were presented with a yellow light for 4 s, followed by a reward, independent of their behavior. On other trials, they were presented with a blue light for 4 s but it was not followed by a reward, again independent of their behavior. This procedure is sometimes referred to as differential autoshaping [25]. When pigeons are presented with a light that is followed by food, they will often peck at the light, in spite of the fact that pecking is not required, but typically, they will not peck at a light when it is not followed by food [26]. Thus, Zentall et al. [24] proposed that this procedure should result in the pigeon’s expectation that food will follow the presentation of the yellow light but not the blue light. On (unexpected) probe trials, a yellow or a blue light was presented followed by a choice between a red light and a green light. Recall that the presentation of the red and green lights can be considered asking the unexpected question, “What did you just do, did you just peck or did you just refrain from pecking?” Although pecking was not required to the yellow light, it did occur. In no sense, however, would the pigeon be expected to remember that it had pecked, unless it could go back in time and remember that episode and then peck the red light. Similarly, it would not be expected to remember whether it had pecked or not in the presence of the blue light, but if it could go back in time and remember that episode, it would peck the green light. In that experiment, the pigeons chose according to whether they had pecked earlier or not. In a follow-up test [24], the pigeons were presented with a novel circle shape or no stimulus, followed by a red/green test. Although they had never seen the novel circle shape before, the pigeons readily pecked the circle and then after it was turned off, they tended to choose the red light, whereas when the circle had not been presented, they chose the green light.

**Figure 2:**
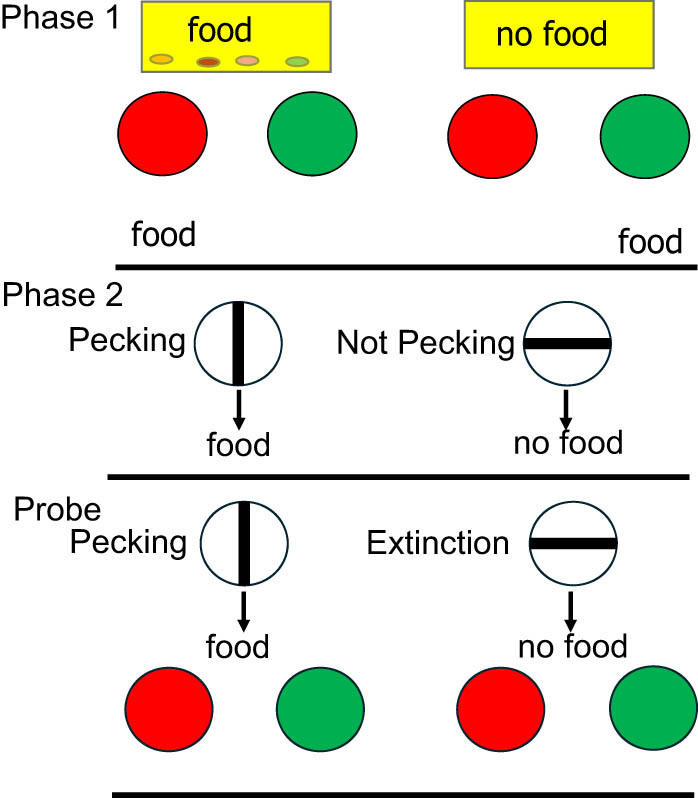
Design of pigeon episodic memory experiment: “Did you get fed or not?”: In Phase 1 after pigeons were fed, they would be fed again for pecking the red light, but if they were not fed, they would be fed for pecking the green light. In Phase 2, vertical line meant pecking was followed by food, horizontal line meant pecking was followed by no food (extinction). On Probe trials, vertical line meant pecking was followed by food, and food was followed by a choice between red and green; horizontal line meant pecking was followed by no food, and no food was followed by a choice between red and green [26].

Using a similar design that came closer to asking the question, what did you have for dinner, it was found that pigeons could retrieve whether they had been fed or not [27]. Pigeons were first trained on a conditional discrimination in which samples of food were followed by a choice between red and green lights (Figure 2). Red was correct if the pigeon had just been fed; green was correct if the pigeon had not been fed. In the second phase of the experiment, the pigeons were trained on a simple successive discrimination in which pecking vertical lines was followed by food but horizontal lines was not. They were then given (unexpected) probe trials in which the vertical/horizontal lines, food/no-food presentations were followed immediately by red and green lights. That is, they were asked *unexpectedly* if they had been fed or not. On those probe trials, the pigeons chose correctly over 66% of the time.

Interestingly, another type of probe trial was also included. They received trials on which the horizontal lines were followed by food and the vertical lines were not (the opposite of what they experienced during original training). Again, choice of the red and green lights followed. Surprisingly, on those probe trials, the pigeons chose correctly, whether they had been fed or not, even more often; over 80% of the time. Although one might have expected poorer accuracy, the fact that the outcome following presentation of the lines was surprising, likely made the outcome more memorable. Thus, the results indicate that when unexpectedly questioned, pigeons can remember what they did earlier and whether they got fed or not earlier.

**Figure 3:**
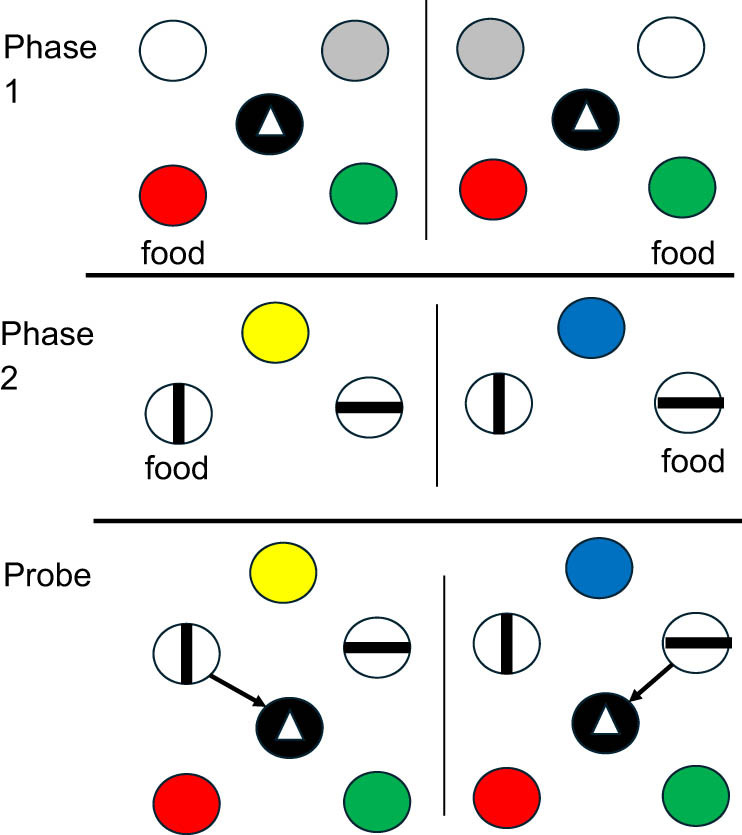
Design of pigeon episodic memory experiment: “Where did you peck?”: In Phase 1, pigeons were trained to peck a single side light then peck the center triangle. If the initial peck was to the left, the red light was correct. If the initial peck was to the right, the green light was correct. Red and green appeared randomly on the left and right. In Phase 2, a yellow light indicated that the choice of the vertical line was correct. A blue light indicated that the choice of the horizontal line was correct. Probe trials were the same as the Phase 2 trials except a peck to the center triangle was followed by a choice of the red or green light [27].

Singer and Zentall [28] explored the unexpected question procedure further by asking if pigeons could remember the location that they pecked when they were unexpectedly asked. In their experiment, the location requested was irrelevant to the task that they had acquired. To achieve this, they first trained pigeons to report the location that they just pecked (Figure 3). Initially, a white light was presented on either the left side or the right side. A peck turned off the white light. Then, to center the pigeon between the two sides, a triangle was presented in the middle, and a peck to the triangle turned on red and green lights on the left and right (counterbalanced over trials). If the initial light had been on the left, a peck to the red light was rewarded. If the initial light had been on the right, a peck to the green light was rewarded.

In Phase 2 of the experiment, the pigeons were trained on a conditional discrimination to choose vertical lines when the sample was yellow and to choose horizontal lines when the sample was blue. The lines were presented randomly on the left and right sides. On unexpected probe trials, after the pigeons had chosen the correct line orientation, a triangle appeared on the center key, and a response to the triangle presented red and green lights on the left and right. Although the location of the line orientations was irrelevant to the conditional discrimination, the pigeons responded correctly to the red and green stimuli, over 71% of the time, as a function of the side on which they had pecked the line orientation.

**Figure 4:**
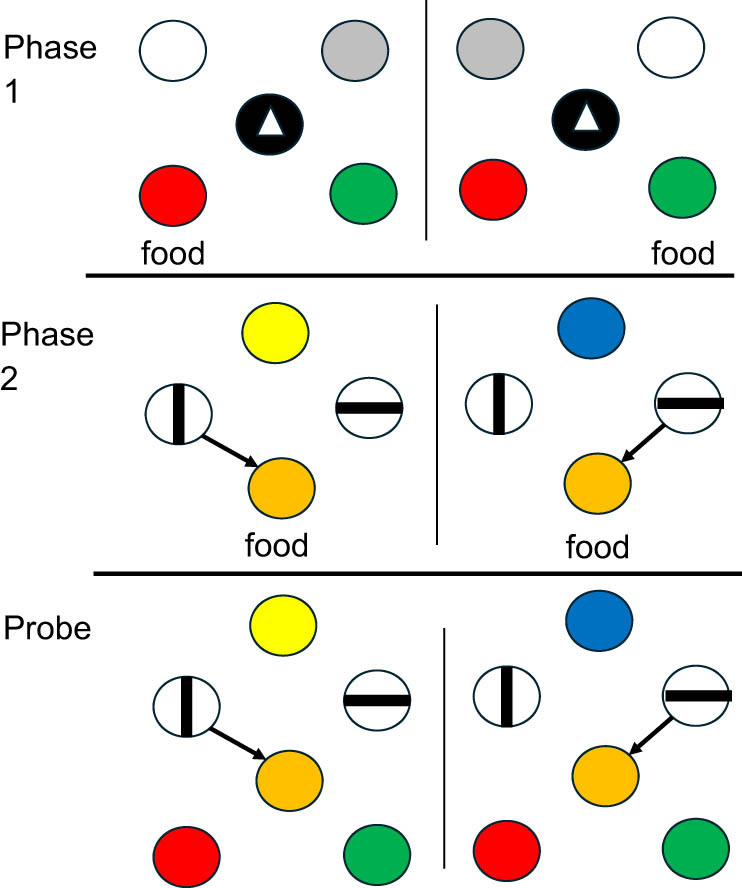
Design of pigeon episodic memory experiment: Where did you peck?”: In Phase 1, pigeons were trained to peck a single side light then peck the center triangle. If the initial peck was to the left, the red light was correct. If the initial peck was to the right, the green light was correct. Red and green appeared randomly on the left and right. In Phase 2, a yellow light indicated that choice of the vertical line was correct and the pigeon was fed after pecking the center orange light. A blue light indicated that choice of the horizontal line was correct and the pigeon was fed after pecking the orange light. Probe trials were the same as the Phase 2 trials except a peck to the orange light was followed by choice of the red or green light [28].

In a follow-up experiment [29], this design was repeated, but to increase the surprise associated with the probe trials, during training on the Phase 2 conditional discrimination, they included the center key response that would occur on probe trials. That is, in Phase 2, when the pigeons had correctly matched the line orientation comparisons, they had to respond to a stimulus in the center before obtaining their reward (Figure 4). However, in Phase 1, it ensured that the pigeon was oriented to the center, whereas, in Phase 2, it was just an additional response prior to reward. That way, on probe trials, the appearance of the stimulus in the center would not have served as a cue to remember which side response had been made. Once again, the pigeons chose the appropriate color corresponding to the side key that they had pecked over 62% of the time.

## 5 The answer to an unexpected question in rats

**Figure 5:**
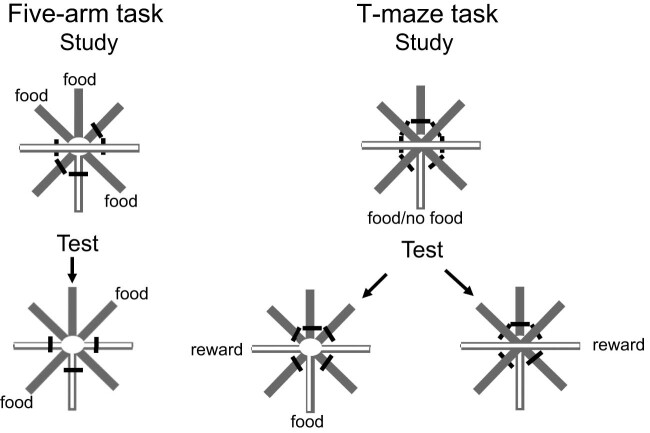
Rats were trained on the five-arm task (top left) with three arms open and were then tested with all five arms open and food in the unentered study arms (bottom left). Rats were also trained on the T-maze task (top right) with food in the stem and were then then tested with or without food in the stem(bottom right). If food in the stem, reward was found in the left arm, if no food in the stem, reward was found in the right arm [29].

**Figure 6:**
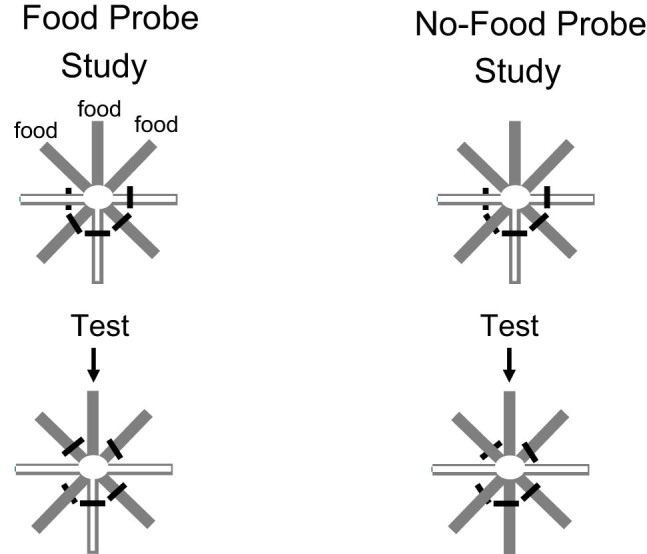
On probe trials, rats were given a five-arm study trial with three arms open. They were then tested on the T-maze. On T-maze food probe trials (left), food was present in the “stem.” On T-maze no-food probe trials (right), food was absent in the “stem” [29].

In an ingenious experiment with rats, Zhou et al. [30] designed a way to test episodic memory involving the unexpected question. Using a radial maze, they first trained rats using five of the arms (omitting the left, right, and bottom arms, see Figure 5). In the initial foraging task, the rats were forced down three of the arms (randomly chosen) to food and shortly after, with food in the remaining two arms, the rats were free to choose from among all five arms,. Meanwhile, the omitted arms that formed a T maze were used in a conditional discrimination, in which food (or the absence of food) in the bottom arm indicated which way to turn (left or right) to get food (or further food). For example, a left turn was rewarded after food was found in the bottom arm, whereas a right turn was rewarded after no food was found in the bottom arm. Finally, on a single probe trial, with three of the arms from the foraging task baited, the rat was then, unexpectedly allowed to choose between the left and right arms (Figure 6). And on another single probe trial, this time with three of the arms from the foraging task *unbaited*, the rat was again, unexpectedly allowed to choose between the left and right arms. The results indicated that on both trials, overall, the rats chose the appropriate left or right arm to a significant degree. That is, when unexpectedly probed, they chose the correct side based on whether they had found food or not from the other task.

Following the research with pigeons and rats, experiments have demonstrated that other species such as dogs [31], bottlenosed dolphins [32], and tits [33] pass the unexpected question test for episodic memory.

**Figure 7:**
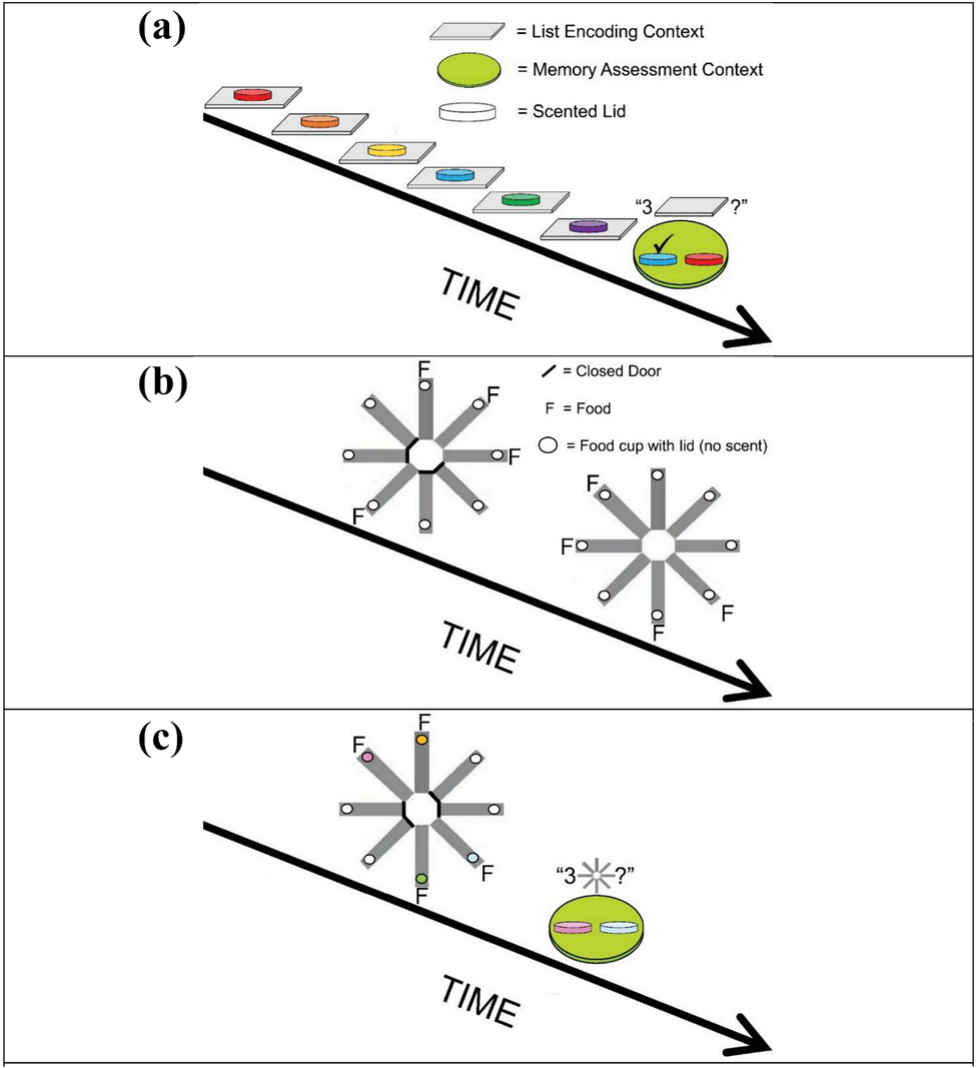
Replay of episodic memory after incidental encoding in an unexpected assessment of memory. (a) Rats were trained to replay episodic memory. After exposure to a sequence of odors of various lengths (4–11), they were tested for their memory of the third one back. (b) Separately, rats foraged for food below unscented lids on a radial maze. (c) Rats foraged for food under scented lids on the radial maze and were then (unexpectedly) prompted to replay memory for the third odor back [30].

Recently, Sheridan et al. [34] identified a characteristic of episodic memory not true of human semantic memories or rule-based memories. They noted that episodic memory typically involves the *replay* of memories that were incidentally encoded. Thus, for example, if a human is asked what they had for dinner last night, if it was an episodic memory, they might consider the sequence of events that led to and accompanied dinner. On the other hand, if they habitually ate the same meal on that day of the week, the answer would likely have come without the additional sequence of events. To encourage the rats to replay memories of events that they had experienced, the researchers presented rats with a sequence of odors of various lengths (4–11 odors), after which the rats were trained to respond selectively to the odor that was the third from the last item in the list they had experienced, rather than to one of the other odors (Figure 7). Because the rats could not know when the list would end, when the rats were tested, it was assumed that the procedure would require that the rats *replayed* the list.

In Phase 2, the rats were trained on an eight arm radial maze, in which after being forced down four of the arms to food, they were allowed to visit the remaining arms, the only ones that were now baited with food. Then, on a single probe trial, after being forced down the four initial arms that for the first time were accompanied by distinctive odors, they were unexpectedly given a two-odor test between the third odor back they had experienced and one of the other odors that they had experienced on the initial four arms. All of the rats chose the third odor back correctly. Thus, when unexpectedly asked which odor they had experienced third back, they were able to replay the recently experienced odors to respond correctly. Furthermore, when the researchers repeated the experiment and in Phase 1 introduced a 15-m delay between the sequence of odors and the two-odor test, the results of the probe trial were essentially the same. Thus, the results indicated that the rats were able to replay the odors under a modest delay, a finding consistent with the view that episodic memory should be a characteristic of long-term memory. The ability of the rats to replay the sequence of odors experienced when unexpectedly asked to do so is strongly suggestive of episodic memory.

The strongest evidence for human episodic memory is the personal experience of metaphorically traveling back in time to recover the memory of an incidental event. The best evidence of episodic memory in other humans is their report of the sequence of events that preceded and accompanied the incidental memory. Although in the absence of language, it would be difficult to find such evidence of episodic memory in nonverbal animals, the rats’ ability to replay recently experienced events when unexpectedly requested to do so has the important characteristic of human episodic memory. When unexpectedly tested, the rat must review the recently presented odors to identify that odor that was presented three back.

Although the absence of language in animals produces unique problems for the studying episodic memory, an alternative approach is to ask if the neural mechanisms known to underly episodic memory in humans are similar to the neural mechanisms that have been found when animals are thought to remember episodically. Functional neuroimaging in humans have shown that episodic memory critically depends on the integrity of the hippocampus [35] but also involves a large network of cortical areas that includes the adjacent parahippocampal region and the prefrontal cortex [36], and there is evidence that this basic memory neurobiology is not unique to humans. That is, there are clear similarities in the circuit organization and functions of these regions across mammals and birds, and especially within mammals [37]. Thus, the neurobiological homology suggests that there are similar pathways capable of supporting episodic memory in humans and other animals.

Another approach to studying episodic memory in nonverbal organisms is to examine the fMRI scans of nonverbal infants. Greater activity in the hippocampus during the viewing of previously unseen photographs was related to later memory-based looking behavior, suggesting that the capacity to encode individual memories is present during infancy [38]. Thus, language does not appear to be necessary for the formation of episodic memories.

One might argue that the experiments with pigeons [24,27,28,29] and even the experiments with rats [30,34] involve a short time between the event and the test for that event, and episodic memory has typically been defined as an example of long-term memory. But is episodic memory necessarily long-term memory? One could imagine the following human scenario: One arrives at one’s front door and cannot find one’s keys where they ought to be. One then mentally retraces one’s steps, usually backward. In doing so, an episodic memory of having been recently distracted by a phone call leads one to return to that location to determine if the keys are there. Thus, the requirement that episodic memory must be shown to involve events that took place some arbitrary time ago may not be necessary. It has been suggested that a better requirement to distinguish an episodic memory from a semantic or rule learning memory should be that it involves an implicit (unintentional) rather than explicit (intentional) memory [39].

## 6 The distinction between recall and familiarity

For humans, one can have a clear memory of an event yet not be able to place it in time. However, it is possible that in asking the unexpected question, such as “what did you have for dinner last night” the question could be answered by judging which memory of food was most familiar, without being able to place the food in time (last evening). In human memory research, a distinction has been made between the recollection of past experiences and judgments of familiarity [40]. For humans, the recollection of past experiences may come with information about the context in which the event appeared (e.g., the item appeared in green font or on the left side of the screen), whereas judgments of familiarity do not generally include contextual information [41].

Alternatively, it has been suggested that recollection of past experiences and judgments of familiarity may be distinguishable by comparing measures of the fMRI at encoding and retrieval [42], and there is evidence that the fMRI of an episodic memory is more similar to the fMRI at original encoding than the fMRI during judgments of similarity [42]. Although such procedures would appear to be promising, it has been argued that attempts to make such a clear distinction between memories based on episodic memory and those based on familiarity using fMRI have not been persuasive [43]. Whether one can make such a distinction in non-verbal animals is not clear, but it would be worthy of study.

With regard to research with pigeons on the unexpected question following an incidental memory, the use of novel probe trials makes it unlikely that associative processes can account for the results found. It is possible, however, that familiarity rather than recollection contributed to memory effects that were found. However, research by Sheridan et al. [34] addresses that issue directly. They demonstrated that rats are able to respond correctly when unexpectedly probed to select the third odor back. In that experiment, they introduced an intervening task in a different context before the unexpected test, thus the accurate choice of the third odor back suggests that simple familiarity was not the basis for choice.

## 7 Planning for the future

If, as noted earlier, an animal could learn over its lifetime without the need for episodic memory, one may ask, what is the function of memory for episodes demonstrated by humans? Why is it important for humans to be able to recall memories concerned with unique, personal past experiences? It is possible that episodic memories help to reproduce the context in which the event took place and that makes them more memorable. That is memories for past events may help establish semantic memories [22].

Alternatively, memory of an episode may be useful in helping to prepare for an uncertain future [44]. That is, the ability to retrieve an episode may allow one to project that memory into the future to help prepare the organism for what is to come. Following this line of reasoning, Raby et al. [45] proposed “To the extent that episodic memory and future planning depend on common processes, the caching behaviour of [scrub jays] should reflect an ability to anticipate future need states [p. 919].” In their study of future planning, scrub jays were fed in a central compartment in the evening, either peanuts or kibble, and they could cache uneaten food in two other compartments, X and Y. Each night, after eating and caching, they were placed in either compartment X or compartment Y and they could learn that if they had been placed in compartment X, in the morning peanuts would be provided to them, but if they had been placed in compartment Y, in the morning kibble would be provided to them. The investigators were interested in whether the jays could anticipate what they would be fed in each compartment in the morning. It was found that the scrub jays cached more kibble in the compartment in which they would be fed peanuts in the morning, and they cached more peanuts in the compartment in which they would be fed kibble in the morning [45]. Thus, scrub jays appear to have the capacity to plan for a future motivational state. Comparable results have been found with apes [46] and ravens [47]. Importantly, these findings are inconsistent with the Bischof–Köhler hypothesis [4], which claims that nonhuman animals cannot anticipate and plan for future needs that they do not currently experience (see [48] for similar claims).

## 8 Conclusions

The study of comparative cognition in which the abilities of humans is compared to those of other animals is made difficult for many reasons. One of the most important among them is the inability of other animals to use anything comparable to human language. This is especially critical when assessing episodic memory because language allows us to differentiate between memory for facts (semantic memory) and memory for experiences (episodic memory). Knowing the *what*, *where*, and *when* of an event is one aspect of episodic memory but it is not sufficient (nor, it can be argued, is it necessary). What makes episodic memory uniquely different from semantic memory is that it typically consists of an incidental event that was not expected to be remembered, such that, for humans, it must be replayed. Thus, to clearly make the distinction, episodic memory should involve the answer to an unexpected question about an incidental event. Several examples of memory processes that have the characteristics of episodic memory have been demonstrated in pigeons [23,27,28] and in rats [13,14,15,29], and an even more convincing procedure involving the replay of events has been demonstrated in rats [34]. Although it may not be possible to obtain in nonhuman animals the kind of evidence for episodic memory and future planning that one can obtain in humans who have access to language, recent research findings with pigeons and rats provide evidence that the cognitive abilities needed to replay a sequence of episodic memories likely predates the emergence of human language.

## References

[1] Tulving E. Episodic and semantic memory. In: Tulving E, Donaldson W, editors. Organization of memory. New York: Academic Press; 1972. p. 381–403.

[2] Vargha-Khadem F, Gadian DG, Watkins KE, Connelly A, Van Paesschen W, Mishkin M. Differential effects of early hippocampal pathology on episodic and semantic memory. Science. 1997 Jul;277(5324):376–80. 10.1126/science.277.5324.376. 9219696

[3] Tulving E. Memory and consciousness. Can Psychol/Psychologie Canadienne. 1985;26(1):1–12. 10.1037/h0080017.

[4] Bischof-Köhler D. Zur phylogenese menschticher motivation [On the phylogeny of human motivation]. In: Eckensberger LH, Lantermann ED, editors. Emotion und reflexivitat. Vienna: Urban & Schwarzenberg; 1985. p. 3–47.

[5] Tulving E, Markowitsch HJ. Episodic and declarative memory: Role of the hippocampus. Hippocampus. 1998;8(3):198–204. 10.1002/(SICI)1098-1063(1998)8:3<198:AID-HIPO2>3.0.CO;2-G. PMID: 9662134. 9662134

[6] Schwartz BL, Colon MR, Sanchez IC, Rodriguez IA, Evans S. Single-trial learning of ‘what’ and ‘who’ information in a gorilla (Gorilla gorilla gorilla): implications for episodic memory. Anim Cogn. 2002;5:85–90. 10.1007/s10071-002-0132-0 12150040

[7] Schwartz BL, Hoffman ML, Evans S. Episodic-like memory in a gorilla: A review and new findings. Learn Motiv. 2005;36:226–44.

[8] Clayton NS, Dickinson A. Episodic-like memory during cache recovery by scrub jays. Nature. 1998;395:272–4. 10.1038/26216 9751053

[9] Clayton NS, Dickinson A. Memory for the content of caches by scrub jays (Aphelocoma coerulescens). J Exp Psychol Anim Behav Process. 1999;25:82–91. 9987859

[10] Clayton NS, Yu KS, Dickinson A. Scrub jays (Aphelocoma coerulescens) form integrated memories of the multiple features of caching episodes. J Exp Psychol Anim Behav Process. 2001;27:17–29. 11199511

[11] Zinkivskay A, Nazir F, Smulders TV. What–where–when memory in magpies (Pica pica). Anim Cogn. 2009;12:119–25. 10.1007/s10071-008-0176-x 18670793

[12] Feeney MC, Roberts WA, Sherry DF. Memory for what, where, and when in the black-capped chickadee (Poecile atricapillus). Anim Cogn. 2009;12:767–77. 10.1007/s10071-009-0236-x 19466468

[13] Zhou W, Crystal JD. Evidence for remembering when events occurred in a rodent model of episodic memory. Proc Natl Acad Sci USA. 2009;106:9525–9. 10.1073/pnas.0904360106 PMC2695044 19458264

[14] Babb SJ, Crystal JD. Discrimination of what, when, and where: implications for episodic-like memory in rats. Learn Motiv. 2005;36:177–89.

[15] Babb SJ, Crystal JD. Discrimination of what, when, and where is not based on time of day. Learn Behav. 2006;34:124–30. 10.3758/bf03193188 16933798

[16] Babb SJ, Crystal JD. Episodic-like memory in the rat. Curr Biol. 2006;16:1317–21. 10.1016/j.cub.2006.05.025 16824919

[17] Roberts WA, Feeney MC, MacPherson K, Petter M, McMillan N, Musolino E. Episodic-like memory in rats: Is it based on when or how long ago? Science. 2008;320:113–5. 10.1126/science.1152709 18388296

[18] Skov-Rackette SI, Miller NY, Shettleworth SJ. What–where–when memory in pigeons. J Exp Psychol Anim Behav Process. 2006;32:345–58. 10.1037/0097-7403.32.4.345 17044738

[19] Iordanova MD, Good MA, Honey RC. Configural learning without reinforcement: integrated memories for correlates of what, where, and when. Q J Exp Psychol. 2008;61:1785–92. 10.1080/17470210802194324 18609388

[20] Clayton NS, Yu KS, Dickinson A. Scrub jays (Aphelocoma coerulescens) form integrated memories of the multiple features of caching episodes. J Exper Psych: Anim Behav Proc. 2001;27(1):17–29. 11199511

[21] Hoffman ML, Beran MJ, Washburn DA. Memory for “what”, “where”, and “when” information in rhesus monkeys (Macaca mulatta). J Exp Psychol Anim Behav Proc. 2009;35:143–52. 10.1037/a0013295 PMC4199307 19364223

[22] Boyle A. Remembering events and representing time. Synthese. 2021;199(1/2):2505–24, https://www.jstor.org/stable/48692956.

[23] Menzel CR. Unprompted recall and reporting of hidden objects by a chimpanzee (Pan troglodytes) after extended delays. J Comp Psych. 1999;113(4):426–34. 10.1037/0735-7036.113.4.426. 10608566

[24] Zentall TR, Clement TS, Bhatt RS, Allen J. Episodic-like memory in pigeons. Psychon Bull Rev. 2001;8:685–90. 10.3758/bf03196204 11848586

[25] Schwartz B. Maintenance of key pecking by response-independent food presentation: the role of the modality of the signal for food. J Exp Anal Behav. 1973 Jul;20(1):17–22. 10.1901/jeab.1973.20-17. PMC1334096 16811689

[26] Brown PL, Jenkins HM. Auto-shaping of the pigeon’s key-peck. J Exp Anal Behav. 1968 Jan;11(1):1–8. 10.1901/jeab.1968.11-1. PMC1338436 5636851

[27] Maki WS. Discrimination learning without short-term memory: Dissociation of memory processes in pigeons. Science. 1979;204:83–5. 10.1126/science.432629 432629

[28] Singer RA, Zentall TR. Pigeons learn to answer the question ‘Where did you just peck?’ and can report peck location when unexpectedly asked. Learn Behav. 2007;35:184–9. 10.3758/bf03193054 17918424

[29] Zentall TR, Singer RA, Stagner JP. Episodic-like memory: Pigeons can report location pecked when unexpectedly asked. Behav Proc. 2008;79:93–8. 10.1016/j.beproc.2008.05.003 18602224

[30] Zhou W, Hohmann AG, Crystal JD. Rats answer an unexpected question after incidental encoding. Curr Biol. 2012;22:1149–53. 10.1016/j.cub.2012.04.040. PMC3376895 22633809

[31] Fugazza C, Pogány Á, Miklósi Á. Recall of others’ actions after incidental encoding reveals episodic-like memory in dogs. Curr Biol. 2016;26(23):3209–13. 10.1016/j.cub.2016.09.057 27889264

[32] Davies JR, Garcia-Pelegrin E, Baciadonna L, Pilenga C, Favaro L, Clayton NS. Episodic-like memory in common bottlenose dolphins. Curr Biol. 2022;32(15):3436–42. 10.1016/j.cub.2022.06.032 35882234

[33] Davies JR, Keuneke LS, Clayton NS, Davidson GL. Episodic-like memory in wild free-living blue tits and great tits. Curr Biol. 2024;34(16):3593–602. 10.1016/j.cub.2024.06.029 38964317

[34] Sheridan CL, Lang S, Knappenberger M, Albers C, Loper R, Tillett B, et al. Replay of incidentally encoded episodic memories in the rat. Curr Biol. 2024 Feb;34(3):641–7.e5. 10.1016/j.cub.2023.12.043. Epub 2024 Jan 12. 38218186

[35] Eichenbaum H, Fortin NJ. Bridging the gap between brain and behavior: Cognitive and neural mechanisms of episodic memory. J Exp Anal Behav. 2005;84(3):619–29. 10.1901/jeab.2005.80-04 PMC1389783 16596982

[36] Cabeza R, St Jacques P. Functional neuroimaging of autobiographical memory. Trends Cogn Sci. 2007;11(5):219–27. 10.1016/j.tics.2007.02.005 17382578

[37] Allen TA, Fortin NJ. The evolution of episodic memory. Proc Natl Acad Sci USA. 2013;110:10379–86, http://www.jstor.org/stable/42706670. 10.1073/pnas.1301199110 PMC3690604 23754432

[38] Yates TS, Fel J, Choi D, Trach JE, Behm L, Ellis CT, et al. Hippocampal encoding of memories in human infants. Science. 2025;387:1316–20. 10.1126/science.adt7570. 40112047

[39] Hampton RR, Schwartz BL. Episodic memory in nonhumans: What, and where, is when? Curr Opin Neurobiol. 2004 Apr;14(2):192–7. 10.1016/j.conb.2004.03.006. PMID: 15082324. 15082324

[40] Mandler G. Recognizing: The judgment of previous occurrence. Psych Rev. 1980;87(3):252–71.

[41] Yonelinas AP. The nature of recollection and familiarity: A review of 30 years of research. J Mem Lang. 2002;46(3):441–517. 10.1006/jmla.2002.2864.

[42] Johnson JD, Rugg MD. Recollection and the reinstatement of encoding-related cortical activity. Cereb Cortex. 2007 Nov;17(11):2507–15. 10.1093/cercor/bhl156. Epub 2007 Jan 4. PMID: 17204822. 17204822

[43] Johnson JD, McDuff SG, Rugg MD, Norman KA. Recollection, familiarity, and cortical reinstatement: a multivoxel pattern analysis. Neuron. 2009 Sep;63(5):697–708. 10.1016/j.neuron.2009.08.011. PMID: 19755111; PMCID: PMC2771457. PMC2771457 19755111

[44] Schacter D, Addis D, Buckner R. Remembering the past to imagine the future: The prospective brain. Nat Rev Neurosci. 2007;8:657–61. 10.1038/nrn2213. 17700624

[45] Raby CR, Alexis DM, Dickinson A, Clayton NS. Planning for the future by western scrub-jays. Nature. 2007;445:919–21. 10.1038/nature05575. 17314979

[46] Mulcahy NJ, Call J. Apes save tools for future use. Science. 2006;312:1038–40. 10.1126/science.1125456 16709782

[47] Kabadayi C, Osvath M. Ravens parallel great apes in flexible planning for tool-use and bartering. Science. 2017;357:202–4. 10.1126/science.aam8138. 28706072

[48] Suddendorf T, Corballis MC. Mental time travel and the evolution of the human mind. Genet Soc Gen Psychol Monogr. 1997;123:133–67. 9204544

